# Supplementary material for: SyNDI: synchronous network data integration framework
Source: BMC Bioinformatics. 2018 Nov 6;19:403. doi: 10.1186/s12859-018-2426-5 (PMC6219086; doi:10.1186/s12859-018-2426-5)
Supplement: Supplementary file 5 — Files of the example presented in Additional file 4. This additional file is a zip package that contains all files used in Additional file 4. Detailed descriptions of each of these files are presented in a README file included in this zip package. (GZ 30411 kb) [file 12859_2018_2426_MOESM5_ESM.gz › SyNDITools/Meme2Fimo/meme_4.9.0_4.tar.gz!/meme_4.9.0/doc/glam2_dirichlet.html]

GLAM2 Dirichlet Mixtures


# GLAM2 Dirichlet Mixtures

## Dirichlet mixture files

A Dirichlet mixture file specifies residues' tendencies to align
with one another, and is the basis for scoring columns of aligned
residues. The format is identical to that of UCSC
Dirichlet mixtures. For examples, see recode3.20comp (copied from UCSC)
and glam\_tfbs.1comp in the GLAM2 examples directory.

The GLAM2 programs only read lines beginning with Mixture= or
Alpha=. Mixture= is followed by a number giving the weight of that
mixture component: these weights should sum to 1. Alpha= is followed by
a list of numbers giving the pseudocounts for that mixture component, as
many as there are symbols in the alphabet. The first number after Alpha=
is the sum of the pseudocounts, and is in fact ignored by the GLAM2
programs.

The pseudocounts should be in the same order as the alphabet
symbols. For the n (nucleotide) alphabet, this is: acgt. For the p
(protein) alphabet, this is: ACDEFGHIKLMNPQRSTVWY.

## Built-in Dirichlet mixtures

If no Dirichlet mixture file is specified, the default is to use
recode3.20comp for the p (protein) alphabet, glam\_tfbs.1comp for the n
(nucleotide) alphabet, and a uniform prior for user-specified alphabets.
